# Blinded SAPS-PD Assessment After 10 Weeks of Pimavanserin Treatment for Parkinson’s Disease Psychosis

**DOI:** 10.3233/JPD-202047

**Published:** 2020-10-27

**Authors:** Stuart H. Isaacson, Bruce Coate, James Norton, Srdjan Stankovic

**Affiliations:** a Parkinson’s Disease and Movement Disorders Center of Boca Raton, Boca Raton, FL, USA; b ACADIA Pharmaceuticals Inc., San Diego, CA, USA

**Keywords:** Efficacy, Parkinson’s disease psychosis, pimavanserin, safety, tolerability

## Abstract

**Background:**

Parkinson’s disease psychosis (PDP) is a common nonmotor symptom that affects up to 60% of patients. Pimavanserin, a selective 5-HT_2A_ inverse agonist/antagonist, is approved for treating hallucinations and delusions associated with PDP.

**Objective:**

Evaluate the efficacy and tolerability of pimavanserin in an open-label extension (OLE) study.

**Methods:**

Patients completing a pivotal 6-week placebo-controlled trial (Core Study) could enroll in the OLE. All patients pimavanserin 34 mg once daily, blinded to previous treatment allocation. Prespecified blinded assessments at Week 4 were the Scale for the Assessment of Positive Symptoms (SAPS) PD version and SAPS H + D scales, Caregiver Burden Scale (CBS), and Clinical Global Impression (CGI) Improvement and Severity scales.

**Results:**

Of 171 who entered the OLE, 148 (87%) completed Week 4. Among patients who received placebo in the Core Study, mean (SD) change from OLE baseline to OLE Week 4 for the SAPS-PD was – 3.4 (6.3); *p* < 0.0001. Mean change from Core Study baseline to OLE Week 4 for SAPS-PD was similar among prior pimavanserin- and placebo-treated patients (–6.9 vs. –6.3). Improvement was similar with CGI-I, CGI-S, CBS, and SAPS-H + D in patients previously treated with placebo. Adverse events occurred in 92 (53.8%) patients during the 4-week OLE.

**Conclusion:**

Improvements at OLE Week 4 from pretreatment baseline were similar with placebo and pimavanserin in the Core Study. The beneficial effects observed with pimavanserin in the 6-week Core Study were maintained for 4 weeks in the blinded OLE, supporting the durability of response with pimavanserin 34 mg for PDP over 10 weeks.

## INTRODUCTION

Hallucinations and delusions are common neuropsychiatric symptoms in patients with Parkinson’s disease (PD), developing in over 50% of patients over the course of their disease with progression over time [1–3]. The onset of PD psychosis may be insidious and recognition may be delayed. PD psychosis complicates management of motor symptoms by limiting increases of dopaminergic medications, and its symptoms can result in anxiety, depression, stigma, social withdrawal, and increased caregiver burden [2]. Parkinson’s disease psychosis (PDP) is a major risk factor for hospitalization, prolonged hospitalization, nursing home placement, and mortality [4–8]. Treatment of PDP has relied on identifying provoking systemic illnesses, and minimizing anticholinergic and other offending medications. However, limiting dopaminergic therapy (i.e., not increasing when needed for motor symptoms for fear of worsening psychosis symptoms, or reducing medications with worsening motor symptoms) may have the consequence of increasing disability and morbidity. Prior to the approval of pimavanserin the only antipsychotics that did not worsen motor symptoms involved the off-label use of quetiapine or clozapine. All other antipsychotics typically worsen Parkinsonism through their blockade of postsynaptic D_2_ receptors, and MDS guidelines and Beers criteria caution against their use [9]. Recent guidelines from the MDS state that quetiapine has insufficient evidence with an acceptable risk and possibly clinically useful, and clozapine as efficacious but requiring specialized monitoring and clinically useful [10].

The selective 5-HT_2A_ receptor inverse agonist, pimavanserin, is devoid of dopaminergic, histaminergic, adrenergic or muscarinic activity [11]. In a Phase 3, placebo-controlled study, pimavanserin 34 mg improved hallucinations and delusions associated with PDP, without worsening motor symptoms [12]. Perhaps related to the absence of other receptors affinities, daytime somnolence, orthostatic hypotension, and constipation were not identified as common adverse effects [12, 13]. Pimavanserin also improved daytime sleepiness and nocturnal sleep, and reduced caregiver burden over the 6-week treatment period [12]. Pimavanserin received regulatory approval by the FDA in the U.S. for the treatment of hallucinations and delusions associated with PDP in April 2016.

Patients who completed a double-blind, placebo-controlled, 6-week (Core) study were able to enroll in a long term open label extension (OLE) study, which included a prespecified SAPS-PD assessment at OLE Week 4. This prespecified prospective analysis was evaluated in patients who either continued treatment with pimavanserin 34 mg for 10 weeks (i.e., randomized to pimavanserin arm in Core study) or began treatment with pimavanserin 34 mg (i.e., had been randomized to placebo in the Core study) for 4 weeks.

## MATERIALS AND METHODS

The study was conducted according to the ethical principles of Good Clinical Practices, the International Council for Harmonisation of Technical Requirements for Pharmaceuticals for Human Use; United States Code of Federal Regulations; and World Medical Association-Declaration of Helsinki. Institutional Review Board or Ethics Committee approval for the protocol and the informed consent Form was obtained at each clinical site. Written approval of these documents was obtained from each patient and caregiver before any study procedures were performed. This study was registered at clinicaltrials.gov: NCT00550238.

### Study design

Participants who completed the randomized, placebo-controlled 6-week pivotal trial (Core Study) were eligible to enter an open-label extension (OLE) trial to assess long-term durability and safety of pimavanserin treatment [14].

This paper reports the analysis of participants who completed the Core study and subsequently entered in the OLE. Clinical sites in North America enrolled patients in the OLE after completion of treatment with 34 mg pimavanserin or placebo for 6 weeks in the Core study (ACP-103-020, NCT01174004). During the initial 4 weeks of the OLE patients and site investigators remained blinded to the original treatment allocation in the Core study. Participants were evaluated at Week 4 using the primary endpoint of the blinded, placebo-controlled pivotal trial (i.e., SAPS-PD), as well as with SAPS-H + D, CBS, CGI-S and CGI-I. Additionally safety assessments were conducted at each study visit.

Baseline assessments were collected from patients and caregivers. For patients who were enrolled >1 week after completing the Core study, baseline assessments were completed at study entry. For patients who enrolled within 1 week of completing the Core study, baseline assessments were captured from those performed at the final Core study visit. A caregiver was required to accompany the patient to all visits, to provide information to study staff regarding the patient’s symptoms, and to complete a questionnaire to assess caregivers’ quality of life (Caregiver Burden Scale) [15]. Following baseline assessments, pimavanserin 34 mg was ingested orally by the patient once daily. Patients and site investigators remained blinded to the original treatment allocation from the Core study.

### Patient selection

Patients who had completed the Core study within the past 28 days were eligible if the Investigator determined they could benefit from continued treatment with pimavanserin. Patients were required to be oriented to time, person, and place. All patients were required to provide informed consent or to have a caregiver who could provide informed consent on behalf of the patient. The caregiver had to agree to accompany the patient to all study visits. Women had to be of non-childbearing potential during the study or agree to use a clinically acceptable method of contraception during the study. Patients were required to have psychotic symptoms of at least moderate severity consistent with established diagnostic criteria for PDP [16]. Improvement of symptoms during the previous Core study was not required for entry into the OLE.

Patients were excluded for any clinically significant medical illness that might interfere with the conduct of the study; use of any prohibited or restricted medications; current use of medications known to prolong the QT interval; a baseline electrocardiogram (ECG) with Bazett’s corrected QT > 460 msec for males or >470 msec for females; or allergy or sensitivity to pimavanserin or other drugs of the same class.

### Study assessments

Symptoms of psychosis were measured on Hallucination and Delusion subscales of the Scale for the Assessment of Positive Symptoms (SAPS) in North America by central, blinded, independent raters (MedAvante, Inc.) through remote video connection and outside North America by qualified off-site blinded raters. The SAPS-PD (modified 9-item SAPS hallucinations and delusions subscales) [17] and the SAPS-H + D (combined 20-item SAPS hallucinations and delusions subscales) [18] were evaluated at Week 4. The Clinical Global Impression-Severity and –Improvement (CGI-S and CGI-I) scales [19] and the Caregiver Burden Scale (CBS) [15] were scheduled after 2 weeks (±3 days) and 4 weeks (±3 days). Unscheduled study visits were allowed at any time. Patients who terminated the study at any time other than a planned study visit were required to have an end-of-study evaluation (early termination visit). At each study visit, physical and neurological examinations, vital signs (blood pressure and heart rate), standard clinical laboratory tests (chemistry, hematology, urinalysis), 12-lead ECG, and treatment-emergent adverse events (AEs) were assessed.

### Statistical analysis

No formal statistical analysis was conducted for the clinical rating scales. Observed mean values and standard deviation were summarized for baseline and the 4 week visit of the OLE. Descriptive statistics were reported including number of patients, mean, median, standard deviation (SD), standard error of the mean (SE), minimum and maximum for continuous measurements and number and percentage of patients in each level of a categorical measurement. *P*-values were unadjusted for multiple comparisons.

## RESULTS

**Fig. 1:**
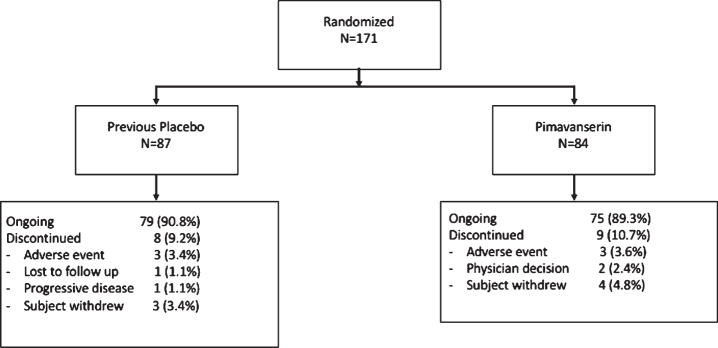
Disposition of patients from Core study eligible for the OLE study. Two patients were in screening for Study 020 when enrollment was closed and were offered open-label treatment in Study 015.

Patient data were collected between August 2010 and December 2012 from 66 clinical sites in North America. For the subgroup of patients that entered the OLE, this report summarizes the full 10 weeks of blinded treatment (6 weeks double blind plus the first 4 weeks of the open–label study). Of 176 patients who completed the ACP-103-020 study and were eligible to enroll, 171 entered the open-label extension (Fig. 1), and 154 (90.1%) patients remained in the study at the Week 4 time point (Table 1).

**Table 1:** Baseline characteristics of study population at enrollment in the Core Study and OLE study

Variable	Core Baseline	OLE Baseline		
	PIM 34 (N = 171)	Placebo-PIM 34	PIM 34-PIM 34	PIM 34
		(N = 84)	(N = 87)	(N = 171)
Age, years^a^	72.6 (7.3)	72.6 (7.9)	72.6 (6.6)	72.6 (7.3)
Age range	53–90	53–90	56–85	53–90
Age category, *n* (%)
<65 years	19 (11.1)	9 (10.7)	10 (11.5)	19 (11.1)
65–75 years	96 (56.1)	48 (57.1)	48 (55.2)	96 (56.1)
>75 years	56 (32.7)	27 (32.1)	29 (33.3)	56 (32.7)
Male, *n* (%)	104 (60.8)	48 (57.1)	56 (64.4)	104 (60.8)
Female, *n* (%)	67 (39.2)	36 (42.9)	31 (35.6)	67 (39.2)
Race, n (%)
White	161 (94.2)	79 (94.0)	82 (94.3)	161 (94.2)
Black or African American	2 (1.2)	1 (1.2)	1 (1.1)	2 (1.2)
Asian	0	0	0	0
Other	8 (4.7)	4 (4.8)	4 (4.6)	8 (4.7)
Height, cm^a^	169.1 (11.1)	168.2 (11.7)	169.9 (10.7)	169.1 (11.2)
Weight, kg^a^	75.4 (17.0)	73.9 (17.4)	76.8 (16.2)	75.4 (16.8)
BMI, kg/m^2a^	26.2 (4.7)	26.1 (4.7)	26.4 (4.7)	26.2 (4.7)
SAPS-PD ^a^	15.2 (5.8)	12.0 (7.3)	9.7 (7.1)	10.9 (7.3)
SAPS-H + D^a^	16.5 (7.1)	12.8 (8.3)	10.6 (8.2)	11.7 (8.3)
CGI-S^a^	4.3 (0.9)	3.9 (1.3)	3.2 (1.3)	3.5 (1.3)

^a^mean (standard deviation).

At OLE baseline, mean age was 72.6 years, 94.2% were white, and 60.8% were male; 88.8% of patients were at least 65 years and 32.7% were over 75 years of age (Table 2). Core study baseline mean SAPS-PD, SAPS-H + D, and CGI-S scores for patients previously on 34 mg pimavanserin were 15.9, 17.6, and 4.3, respectively and for those previously on placebo were 14.4, 15.4, and 4.3, respectively. Participants had a 120.7 month mean duration of PD with a mean 33.2 month duration of PDP. Demographic characteristics for patients entering the OLE were similar to the total population that entered the Core Study (Table 2).

**Table 2:** Adverse events occurring in the first 4 weeks of the OLE grouped by placebo controlled study treatment group (safety analysis set)

Type of Event	Number (%) of Patients			
	All Core Study	Placebo	PIM 34 mg	All
	(*N* = 171)	(*N* = 84)	(*N* = 87)	(*N* = 171)
≥1 AE	112 (65.5)	49 (58.3)	43 (49.4)	92 (53.8)
≥1 Drug-Related AE	30 (17.5)	20 (23.8)	12 (13.8)	32 (18.7)
≥1 SAE	4 (2.3)	2 (2.4)	0 (0.0)	2 (1.2)
AE Leading to Study	0 (0.0)	7 (8.3)	7 (8.0)	14 (8.2)
Termination or Dose
Discontinuation

### Durability of antipsychotic response

**Fig. 2:**
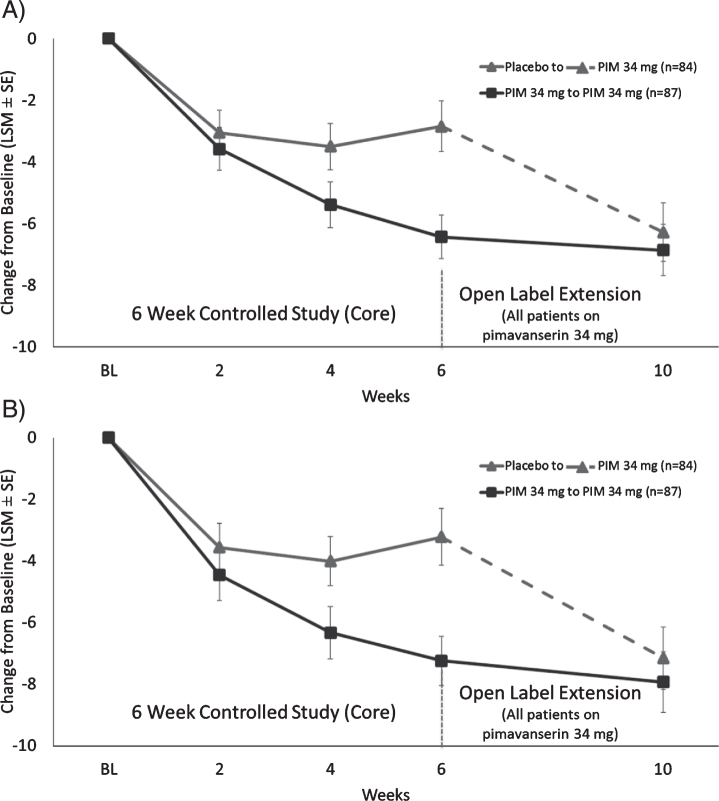
LS mean (SE) change from baseline for A) SAPS-PD score and B) SAPS H + D for patients who were in Study 020 and entered Study 015.

At OLE Week 4 (10 weeks total treatment duration), mean (SD) change from Core study baseline for the blinded SAPS-PD score was similar among prior pimavanserin and prior placebo-treated patients (–6.86 vs. –6.28) (Fig. 2A). Among patients entering the study having received placebo in the Core study, the mean (SD) change from OLE baseline to Week 4 blinded SAPS-PD score was –3.43 (6.3) *p* < 0.0001. For patients previously dosed with pimavanserin 34 mg during the Core study, improvement from Core Study baseline was durable at Week 10 with a mean (SD) change from OLE baseline to Week 4 for the blinded SAPS–PD score of –0.43 (6.8). Mean (SD) SAPS–H + D scores decreased from OLE baseline to Week 4 in the overall population [–2.3 (7.5)], in those receiving prior placebo [–3.88 (7.0) *p* < 0.0001], and in those receiving prior pimavanserin 34 mg remained improved [–0.69 (7.6) *p* = 0.44] (Fig. 2B).

Participants with prior placebo in Core Study experienced improvement from OLE baseline in the mean SAPS-H score at Week 4 of –2.45 (4.8), *p* < 0.0001; patients in the prior pimavanserin 34 mg group remained improved with a mean change of –0.18 (5.5). Patients with prior placebo had a mean change from baseline in the SAPS-D score at Week 4 of –1.43 (4.0) *p* = 0.0027, and patients previously dosed with pimavanserin 34 mg had a mean change from OLE baseline of –0.51 (3.3) *p* = 0.18. Overall, improvement in the SAPS-H and SAPS-D scores that was observed during the Core Study treatment persisted through Week 4 of open-label treatment, while scores improved among patients switched from placebo to pimavanserin.

**Fig. 3:**
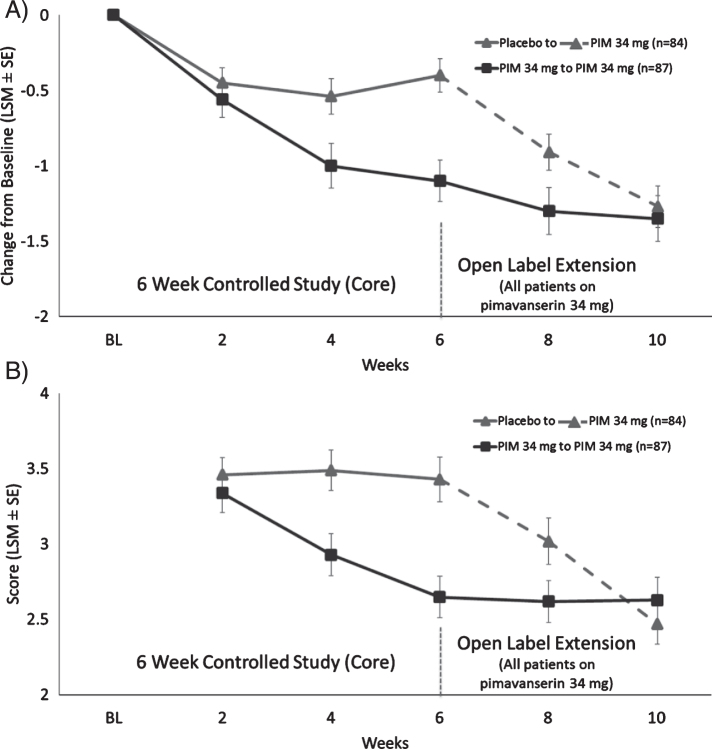
LS mean (SE) change from baseline for A) CGI-S score and B) CGI-I for patients who were in Study 020 and entered Study 015.

For all patients, the mean (standard error [SD]) CGI-S score at Core study baseline was 4.3 (0.9) and at OLE baseline was 3.9 (1.3) denoting mild symptoms. The mean change from OLE baseline to OLE Week 4 for the CGI-S regardless of previous treatment was –0.54 (1.1); the greatest improvement was in patients previously on placebo (–0.86 [1.1] *p* < 0.0001). Patients previously on pimavanserin had a modest improvement in the CGI-S (–0.24 [1.0] *p* = 0.04) indicating that the improvement over baseline seen in the Core Study was increased during the first 4 weeks of the OLE (Fig. 3A).

**Fig. 4:**
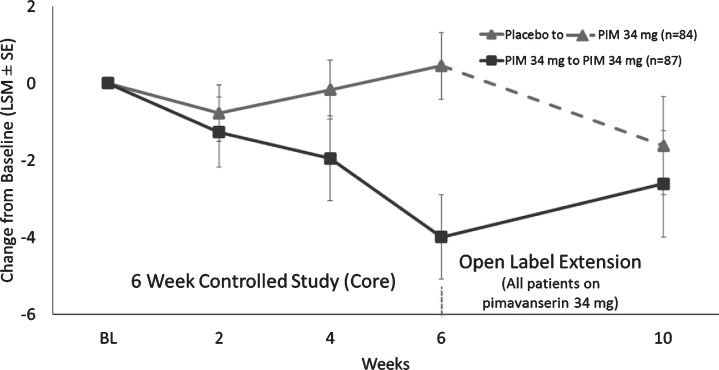
LS mean (SE) change from baseline for Caregiver Burden score for patients who were in Study 020 and entered Study 015.

For CGI-I, the mean (SD) score at Week 2 and Week 4 of the OLE was 2.8 (1.3) and 2.6 (1.2), respectively. For patients previously on placebo, the mean (SD) CGI-I improvement from OLE baseline at Week 2 and Week 4 of the OLE was 2.9 (1.3) and 2.5 (1.2), respectively, while patients previously on pimavanserin remained stable CGI-I (SD) at Week 2 and Week 4 of the OLE with scores of 2.5 (1.1) and 2.6 (1.3), respectively. The proportion of CGI-I responders (very much improved or much improved) was 46.6% at Week 2 and 57.1% at Week 4 (Fig. 3B). The mean CBS score remained stable during the OLE. For patients previously on placebo the change from Core Baseline in CBS (SE) at Week 4 of the OLE was –1.62 (1.3), while patients previously on pimavanserin remained stable with the change from Core study baseline in CBS (SE) at Week 4 of the OLE of –2.6 (1.4) (Fig. 4).

### Tolerability

Following 4 weeks (10 weeks total therapy) AEs causing discontinuation were reported by 6 (3.5%) patients (Table 2). The majority of AEs were of mild or moderate intensity, but 2 (1.2%) patients had serious AEs. The most common AEs were fall (7.0%), hallucination (3.5%), urinary tract infection (5.8%), and peripheral edema (2.9%) (Table 3). The incidence of drug-related AEs during OLE was 23.8% for previous placebo-treated patients and 13.8% for previous pimavanserin-treated patients No clinically relevant changes were observed with pimavanserin for serum chemistry, hematology or urinalysis or ECG findings including no clinically relevant changes in QTc interval.

**Table 3:** Treatment-emergent adverse events occurring ≥2% in all groups combined in the first 4 weeks of the OLE (safety analysis set)

Type of Event	Number (%) of Patients			
	All Core Study	Placebo	PIM 34 mg	All
	(*N* = 171)	(*N* = 84)	(*N* = 87)	(*N* = 171)
Fall	17 (9.9)	5 (6.0)	7 (8.0)	12 (7.0)
Urinary tract infection	17 (9.9)	7 (8.3)	3 (3.4)	10 (5.8)
Insomnia	8 (4.7)	4 (4.8)	3 (3.4)	7 (4.1)
Hallucination	3 (1.8)	4 (4.8)	2 (2.3)	6 (3.5)
Oedema peripheral	9 (5.3)	3 (3.6)	2 (2.3)	5 (2.9)
Excoriation	4 (2.3)	3 (3.6)	1 (1.1)	4 (2.3)
Agitation	0 (0.0)	1 (1.2)	3 (3.4)	4 (2.3)
Confusional state	4 (2.3)	0 (0.0)	4 (4.6)	4 (2.3)
Psychotic disorder	1 (0.6)	2 (2.4)	2 (2.3)	4 (2.3)

## DISCUSSION

During the OLE trial, participants completing the Core study underwent a prespecified, blinded, remote evaluation of the primary endpoint (SAPS-PD) used in the pivotal PDP trial after 4 weeks of open-label treatment. For participants who had received pimavanserin during the Core study, durability of improvement on SAPS-PD was demonstrated after 10 weeks total of pimavanserin treatment without additional safety concerns. Participants entering the OLE who had been randomized to the placebo arm in the Core study had similar improvement in the SAPS-PD after 4 weeks of open-label pimavanserin treatment, beginning after 2 weeks. Among those who switched from placebo to pimavanserin for the OLE, mean scores improved to the same level as the pimavanserin group over the next 4 weeks. This treatment effect was also seen for SAPS H + D, and improvement was maintained for CGI-I, CGI-S, and CGI-I response rates among patients who continued on pimavanserin. No new or unexpected adverse events were recorded.

In this OLE study, the mean change in SAPS-PD at OLE Week 4 was –3.4 points among patients on placebo in the Core study; this was comparable to the treatment effect observed for pimavanserin 34 mg over placebo in 6-week blinded studies of pimavanserin [12, 20]. The SAPS-PD scale retains the reliability, sensitivity to change, and effect size of the larger SAPS-H + D, with reduced score variability. Regression analyses using the SAPS-PD scale indicated that a clinically meaningful change in the CGI-I scale was associated with a 2.33-point change in the SAPS-PD score [17]. Thus, the results obtained in this OLE are consistent with a clinically meaningful improvement.

Prior to the availability of pimavanserin, clinical approaches for PDP treatment have relied on a reduction of dopaminergic medications, a strategy that can lead to worsening of motor symptoms and which also may not be effective in reducing PDP symptoms [9]. The off-label use of antipsychotics was needed. Since all prior antipsychotics are postsynaptic D_2_ receptor antagonists, all typical and atypical antipsychotics worsen motor symptoms in patients with PD except for quetiapine and clozapine. Indeed, MDS guidelines and Beers Criteria caution against the use of all except these two antipsychotics [21, 22]. However, clozapine is rarely used due to blood monitoring for agranulocytosis [9], and despite anecdotal experience the evidence supporting the efficacy and safety of quetiapine remains mixed with sedation and neuroleptic sensitivity common [23–28]. Anecdotal use of cholinesterase inhibitors and other medications have also been reported with varying success [29]. The availability of pimavanserin, an atypical antipsychotic targeting only serotonergic (i.e. 5–HT_2A_) receptors, without antagonism of dopaminergic and other receptors, is an important addition to the treatment paradigm for patients with PDP. Pimavanserin and clozapine are the only two antipsychotics that have demonstrated efficacy in relatively large, blinded, placebo-controlled trials without worsening motor symptoms [12, 30]. Durability of clinical improvement of PDP with pimavanserin after ten weeks of treatment provides additional information on this novel antipsychotic.

Limitations of this study were its open-label design and the lack of a comparison group. Another limitation is selection bias that could have resulted from the non-random selection of patients for the OLE. However, the blind was maintained amongst patients, caregivers, investigators, and remote raters. These results provide the first efficacy and safety information beyond six weeks seen in the pivotal trial with pimavanserin in patients with PDP. These results reinforce that the treatment response observed during double-blind, randomized studies with pimavanserin is maintained during continued treatment for a total of 10 weeks.

Overall, the durability of response, and a sustained improvement in the severity of psychotic symptoms, as assessed with the SAPS-PD, Caregiver Burden, and CGI scales, was demonstrated in patients with PDP receiving treatment with pimavanserin 34 mg daily for 10 weeks.

## CONFLICT OF INTEREST

SI: Honoraria for CME, consultant, research grants, and/or promotional speaker on behalf of: Abbvie, Acadia, Acorda, Adamas, Addex, Allergan, Amarantus, Axovant, Biogen, Britannia, Eli Lilly, Enterin, GE Healthcare, Global Kinetics, Impax, Intec Pharma, Kyowa, Lundbeck, Michael J. Fox Foundation, Neurocrine, Neuroderm, Parkinson Study Group, Pharma2B, Roche, Sanofi, Sunovion, Teva, UCB, US World Meds, Zambon.

BC, JN, and SS were employees of ACADIA Pharmaceuticals, Inc. and may have owned stock in the company.
